# Familiarization: A theory of repetition suppression predicts interference between overlapping cortical representations

**DOI:** 10.1371/journal.pone.0179306

**Published:** 2017-06-12

**Authors:** Giacomo Spigler, Stuart P. Wilson

**Affiliations:** 1 Sheffield Robotics, The University of Sheffield, Sheffield, United Kingdom; 2 Department of Psychology, The University of Sheffield, Sheffield, United Kingdom; Universidad de Salamanca, SPAIN

## Abstract

Repetition suppression refers to a reduction in the cortical response to a novel stimulus that results from repeated presentation of the stimulus. We demonstrate repetition suppression in a well established computational model of cortical plasticity, according to which the relative strengths of lateral inhibitory interactions are modified by Hebbian learning. We present the model as an extension to the traditional account of repetition suppression offered by sharpening theory, which emphasises the contribution of afferent plasticity, by instead attributing the effect primarily to plasticity of intra-cortical circuitry. In support, repetition suppression is shown to emerge in simulations with plasticity enabled *only* in intra-cortical connections. We show in simulation how an extended ‘inhibitory sharpening theory’ can explain the disruption of repetition suppression reported in studies that include an intermediate phase of exposure to additional novel stimuli composed of features similar to those of the original stimulus. The model suggests a re-interpretation of repetition suppression as a manifestation of the process by which an initially distributed representation of a novel object becomes a more localist representation. Thus, inhibitory sharpening may constitute a more general process by which representation emerges from cortical re-organisation.

## Introduction

The more often we encounter an object, for example the more often we see a particular face or hear a particular voice, the more familiar it becomes. The first time we see a new face or hear a new voice, it evokes a *distributed* pattern of activity amongst neurons that otherwise participate in representing faces or voices with which we are already familiar. However, responses to familiar objects are usually more *localized*, to different degrees of sparsity and selectivity [1–3]. Here we investigate how patterns of neural activity change as a novel object becomes familiar.

A distributed representation may be recovered from the responses in a population of neurons, as a linear combination of the features to which those neurons typically respond, weighted by their activities. For example, population activity in cat motor cortex has been found to represent the 3D location of the paw as a vector sum of the paw locations preferred by the individual active neurons [4]. A similar type of distributed representation has been found to encode complex shapes in primate visual area V4 [5], and to encode objects as a combination of simpler features and smaller parts in primate temporal cortex [6]. In general, areas representing complex objects like faces might use a population representation, constructed as the joint activity of neurons selective to similar objects or their parts (e.g., in representations of faces, neurons that are selective to specific eyes, mouths etc.).

If a novel object first evokes a distributed pattern of cortical activity amongst many neurons, then familiarization may correspond to a transition from an initial distributed representation to a more localist representation that involves the activity of a smaller subset of the original population.

This intuitive account of familiarization is indirectly supported by observations of *repetition suppression*, whereby repeated presentations of a stimulus reduce subsequent cortical responses to that stimulus [7]. Repetition suppression has been demonstrated using fMRI, EEG, and single-neuron recordings, in humans and many other mammals [8–12], and it can be modulated by short-term neural habituation [13], synchrony [14], expectation [11], and attention and task-dependency [12, 15]. The opposite effect, *repetition enhancement*, can also be measured, especially at the level of single neurons, with suppression following shortly afterwards [16].

A plausible account of repetition suppression is offered by the *sharpening theory* [17–20], according to which a reduction in cortical activity reflects a narrowing of neuronal tuning curves and a silencing of the responses of the neurons least tuned to the stimulus. The assumptions of sharpening theory have been made explicit in a computational model [21], in which synaptic weights in the *afferent* projections into a cortical network are modified by Hebbian learning, while neurons compete laterally to represent a given input pattern under a simple winner-take-all (k-WTA) operation. The architecture of this model is consistent with a broad range of ‘self-organising network’ models that use similar local competitive learning mechanisms to explain the emergence of continuous topological map patterns resembling those measured in primary cortical areas [22–25].

Here we use a model with explicit Hebbian-modifiable lateral interactions [26] to investigate repetition suppression. The model accounts for the reduction in evoked cortical activity as a strengthening of lateral inhibitory interactions. Essentially, the more often a stimulus is presented the stronger the lateral inhibitory interactions between the responding neurons become, leading to an increase in the selectivity and a reduction in the spatial extent and magnitude of the response. The assumptions of this model are broadly consistent with those of sharpening theory, but the simulations presented herein suggest that plasticity in cortical afferents plays only a secondary role. Indeed, lateral plasticity alone is sufficient to account for repetition suppression. We show how this account can be falsified, by deriving a non-intuitive prediction from the model; repetition suppression for an ‘adapter’ object can be *disrupted* by intervening exposure to objects that produce activity that overlaps with that elicited by the adapter (i.e., by objects that have parts in common with the adapter).

A key prediction of the model is therefore that overlapping cortical representations interfere with oneanother. The prediction of interference offered by this account could be useful in interpretting data collected previously in a variety of contexts, such as visual masking [27], and adaptive forgetting [28], as well as in the context of interference between objects of different semantic categories [29]. Moreover, we explain how this modelling prediction helps discriminate between theories of repetition suppression based on Hebbian plasticity and alternative theories, for example based on neural fatigue.

## Materials and methods

To investigate repetition suppression we use the ‘L-model’ of [26], which can be considered an extension of the first model of map self-organisation proposed by von Der Malsburg in 1973 [22].

The 1973 model comprises a sheet of input units that are connected to a sheet of cortical units, separated into excitatory and inhibitory populations. The input units are connected to the cortical units by *afferent* weighted connections, and the cortical units are connected to each other by *lateral* weighted connections that are excitatory over short distances and inhibitory over larger distances. The inputs elicit an initial response in each cortical unit, computed as a weighted sum of its inputs via the afferent connections, which is squashed using a non-linear (e.g., sigmoidal) output function. The initial cortical activation then propagates through the lateral connections, and the net effect of the short-range excitation and long-range inhibition is a dynamic that clusters an initial distributed cortical activation into a pattern of localised ‘activity blobs’. Hebbian plasticity in the afferent connections consolidates these dynamics, such that a similar pattern of input will cause a similar pattern of blobs to emerge in the future. The afferent weights for each cortical unit are normalized by dividing them by the sum of the afferent weights. If the network is presented with many patterns from a set with some underlying statistical structure then the consolidation of the recurrent dynamics through Hebbian plasticity gives rise to a topological map pattern, such that adjacent units develop similar receptive fields (i.e., similar patterns of afferent connectivity) and thus respond selectively to similar patterns. For example, inputs describing a range of image orientations yield orientation preference maps resembling those measured in primate V1.

The L-model explains the emergence of cortical maps according to the same underlying mechanism; Hebbian plasticity, and short-range excitatory and long-range inhibitory recurrent interactions intrinsic to the cortical area [30–33]. For computational efficiency, the cortical unit in the L-model is defined to be a micro-column rather than a neuron, which allows simulation of a single population of cortical units that are each able to excite or inhibit one another to support map self-organisation. The activity of cortical unit *j* at time *t* is given by,

$$\begin{array}{c} \eta_{j}\left(t\right)=\sigma \left(\alpha_{A}\sum_{a}A_{ja}x_{a}+\alpha_{E}\sum_{e}E_{je}\eta_{e}\left(t-\delta t\right)-\alpha_{I}\sum_{i}I_{ji}\eta_{i}\left(t-\delta t\right)\right), \end{array}$$ (1)

where *A* is its set of afferent connection weights, *E* is its set of excitatory weights, *I* is its set of inhibitory weights, and values of *α* are interaction strengths. *σ* is a piecewise-linear output function (see [26] for full details). The L-model then extends the 1973 model by allowing the recurrent weights between cortical units to change according to the same Hebbian rule as for the afferent weights,

$$\begin{array}{c} w_{jk}\left(t\right)=\frac{w_{jk}\left(t-1\right)+\epsilon_{p}\eta_{j}\eta_{k}}{\sum_{p}w_{jp}\left(t-1\right)+\epsilon_{p}\eta_{j}\eta_{p}}, \end{array}$$ (2)

where *w*_*jk*_ may be the weight of an afferent connection (i.e., by setting *w*_*jk*_ = *A*_*jk*_ and *η*_*k*_ = *x*_*k*_), an excitatory connection (i.e., *w*_*jk*_ = *E*_*jk*_), or an inhibitory connection (i.e., *w*_*jk*_ = *I*_*jk*_), *ϵ* is the learning rate, and *p* is an index over the units for which there are corresponding weights in the set *A*, *E*, or *I*.

An *iteration* of the L-model algorithm occurs at integer timesteps (*t* = 1, *t* = 2 etc.), and each iteration involves defining an input pattern, applying Eq 1 to all cortical units *τ* times to allow the dynamics to settle (*δt* = 1/*τ*), applying Eq 2 to modify the weights, and then resetting all activity in the network to zero before the next iteration.

It is important to emphasize that the L-model does not assume that long-range inhibitory interactions are implemented via long-range inhibitory connections in the cortex. Long-range inhibitory interactions may be implemented via long-range excitation of local inhibitory neurons, as is thought to be the case in e.g., V1 for high-contrast visual inputs (see [34, 35]; and see also refs. [36–39]), and in S1 for strong tactile inputs [40, 41]. The architecture of the L-model is deliberately simplified and does not reflect the detailed anatomy of cortical connectivity. Related models with more complex architectures have demonstrated how the more elaborate circuitry in animal cortices could yield similar results [42, 43], but these require many more parameters and more complicated analysis methods. Whether long-range inhibition is implemented by monosynaptic or disynaptic *connections* is not important for the present modelling results, only that *interactions* be net inhibitory at long distances. See [26, 31] for further discussion.

Further elaborations of the algorithm to include biologically plausible mechanisms of homeostatic plasticity (e.g., a dynamic threshold in the output function *σ*) yield maps that match all available data on the patterning, stability, and robustness of (non-rodent) mammalian maps [26]. The ability of Hebbian-modifiable lateral inhibition to explain these data motivates the L-model as a strong theory of cortical plasticity [25, 44].

Repetition suppression has mostly been recorded in ‘higher’ cortical areas [45], which are characterized by large receptive fields and whose afferent input presumably represents stimuli with a degree of invariance to the lower level features represented in primary cortical areas. Our approach is to investigate repetition suppression in higher cortical areas by training the L-model with afferent input patterns that represent the minimal set of assumptions about the underlying network architecture that are required to reveal the effect. Therefore inputs to the L-model are derived from nine ‘input units’, with the activation of each corresponding to the presence of a particular stimulus feature such as a mouth or an eye. Each cortical unit has nine afferent weights *A* corresponding to the nine input units *x*_*i*_ ∈ [0, 1].

In a period of pre-training, 10,000 input patterns, each a vector with a randomly selected component set to 1 and the remaining eight set to values sampled uniform randomly in the range 0 to 0.3, were presented to the network to initialize the cortical sheet with a smooth map-like representation of the (nine-dimensional) input space. Homeostatic plasticity was enabled during this pre-training period to aid the development of continuous maps. However, as homeostatic plasticity is not a component of our account of repetition suppression it was then disabled for the simulations reported herein. The equations of the homeostatic mechanism used in pre-training are taken from ref. [26], and described in full in S1 Text.

A sheet of 48 by 48 cortical units was simulated. Interaction strengths were set to *α*_*A*_ = 2.2, *α*_*E*_ = 1.2, and *α*_*I*_ = 2.3 respectively, and the cortical dynamics were allowed to settle for *τ* = 16 settling steps. Learning rates were *ϵ*_*A*_ = 0.1, *ϵ*_*E*_ = 0, and *ϵ*_*I*_ = 0.3. Note that maps generated by the model are indistinguishable regardless of whether or not plasticity is enabled in lateral excitatory connections (data not shown), hence plasticity in lateral excitatory connections was disabled (*ϵ*_*E*_ = 0) to allow for a clear interpretation of the results in terms of plastic lateral inhibition (see [26, 46]). The full set of model parameters is included as S1 Text. The model was implemented using the Topographica neural map simulator [47].

**Fig 1:**
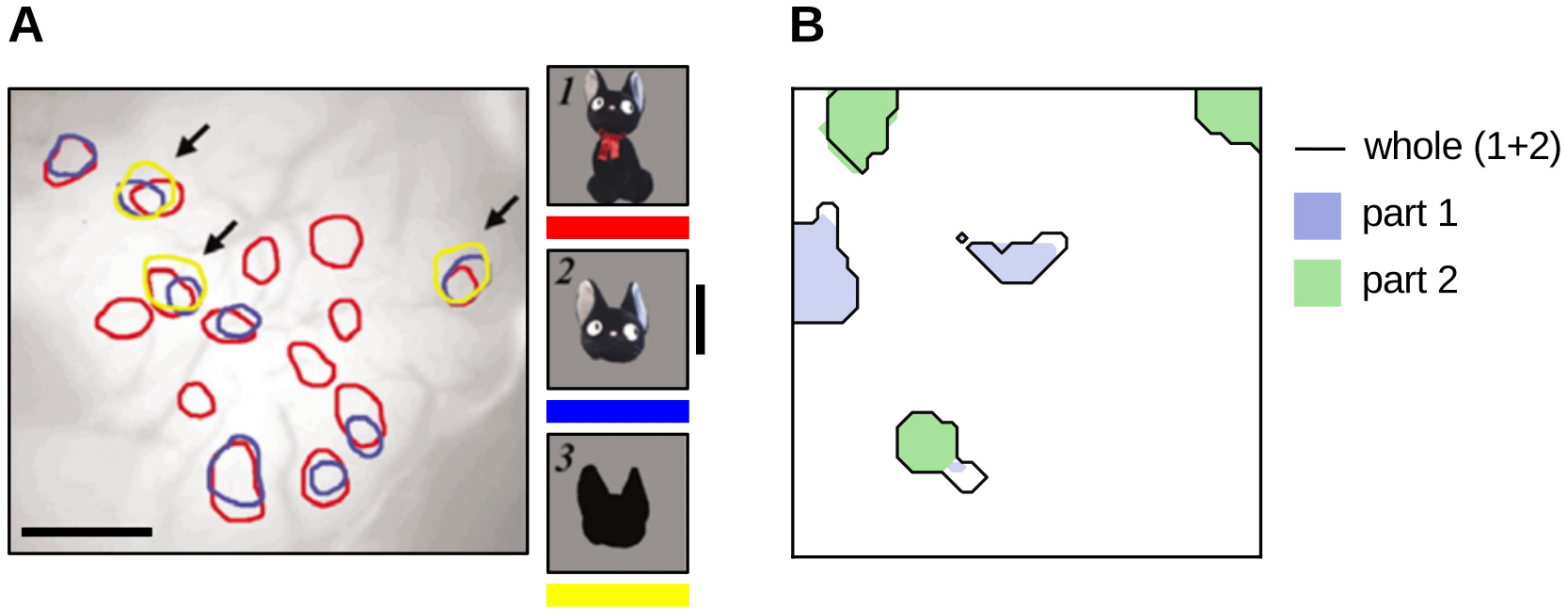
Distributed representation of objects. **A**. Optical imaging of the infero-temporal cortex (IT) of a macaque showing patches of neurons selective to parts of an object. The cortical representation of the whole object significantly overlaps with that of its parts. Adapted from Fig 3b of [6]. **B**. Similar cortical organisation emerges in the L-model of [26] in a simulation of 48 by 48 cortical units. Model units that are active by the object above a threshold of 0.2 are coloured grey, and those which are active by presentation of either of its constituent parts are coloured blue or green.

The mechanistic account of repetition suppression that is provided by the model is best conveyed through plots such as that shown in Fig 1, in which an outline drawn around the units that are active above a threshold of 0.3 highlights the most strongly responsive units. This way the simulation data can be compared with data produced by [6], which shows how the representations of different objects in inferotemporal cortex are distributed and overlapping, and how subsets of the cortical units involved in the representation of complex ‘whole’ objects are selective to the component features. The threshold for visualization was chosen to reveal how the representations learnt by the model are distributed across the network. Note that a high threshold masks some units that participate in a given representation, whereas a low threshold exaggerates the contribution of units with poor stimulus tuning. The thresholded representations shown are thus an approximation used only to help the reader understand the model dynamics, and the extent of the highlighted areas does not necessarily correspond in a one-to-one fashion with the total *response* of the network, which we plot separately in later figures to reveal the dynamics of repetition suppression.

## Results

To investigate how repetition suppression might emerge from intracortical plasticity we ran a set of simulations using the ‘L-model’ [26], according to which both afferent and lateral cortical connectivity is modifiable by Hebbian learning.

### Plastic intracortical connectivity is sufficient to explain repetition suppression

**Fig 2:**
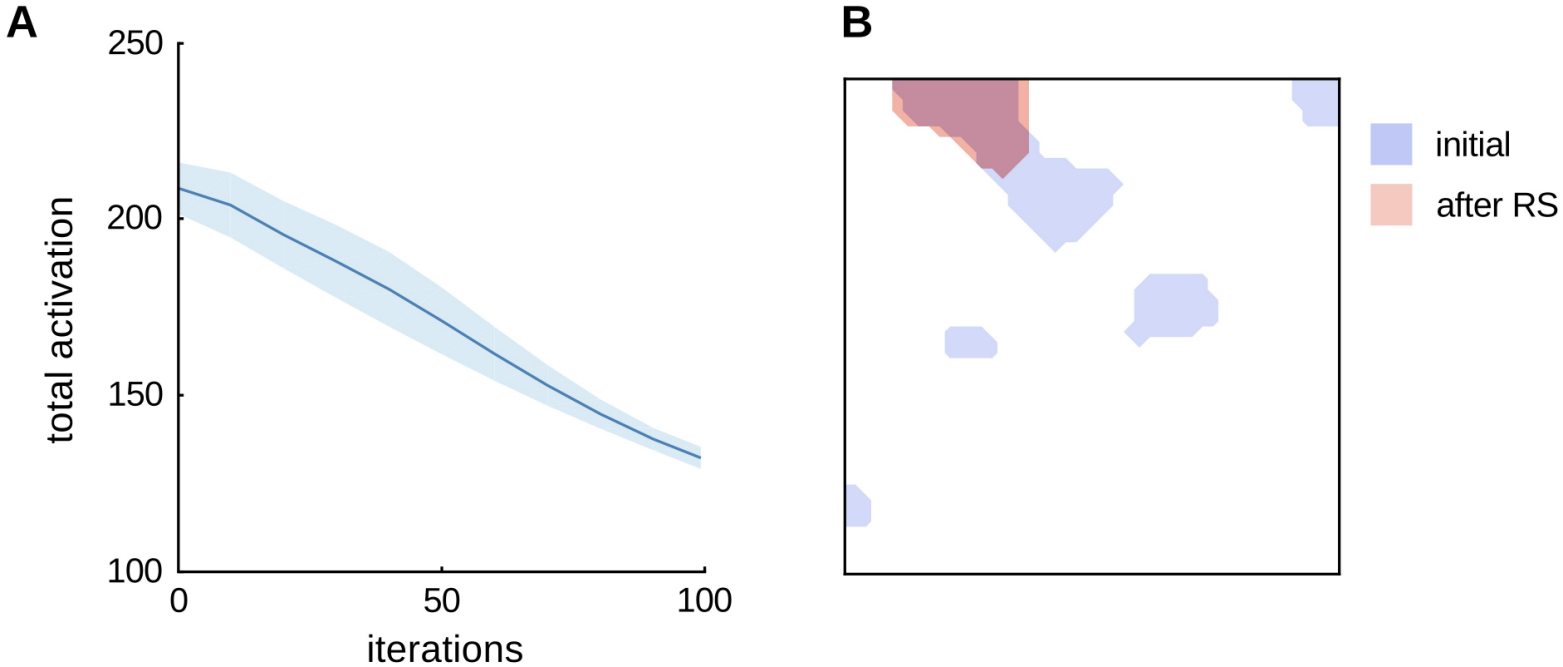
Simulations showing repetition suppression. **A**. The L model [26] shows repetition suppression dynamics when a single input stimulus is presented to the network. The total activation is computed at each iteration as the sum of the activity of all units in the network. The plot is an average of 10 simulations ran with different random initial conditions, with the shaded area representing standard deviation. **B**. The cortical representation of the repeated stimulus is visualized by thresholding the activity of the network before (blue) and after (red) repetition suppression. Representations produced by the model are distributed across stable blobs of highly active units. After repetition suppression, the response is “sharpened” [17], i.e., the sizes of blobs of super-threshold activity shrink.

The first simulation involved presenting the same ‘adapter’ pattern to the network for 100 simulated model iterations, while recording the sum of the activity over all cortical units after the settling of the recurrent dynamics. The adapter was the pattern **x** = {1, 1, 0, 0, 0, 0, 0, 0, 0}, which represents a simple ‘object’ as the configuration of two ‘parts’ (part *x*_1_ and part *x*_2_). The network clearly shows repetition suppression, i.e., a reduction in total cortical activity due to repeated presentation of the stimulus. Inspection of the pattern of activity generated by the network reveals why. Fig 2 presents a comparison of the simulated cortical representation of the adapter stimulus before (blue) and after (red) repetition suppression, in which it is clear that the representation shrinks and ‘sharpens’ over time.

**Fig 3:**
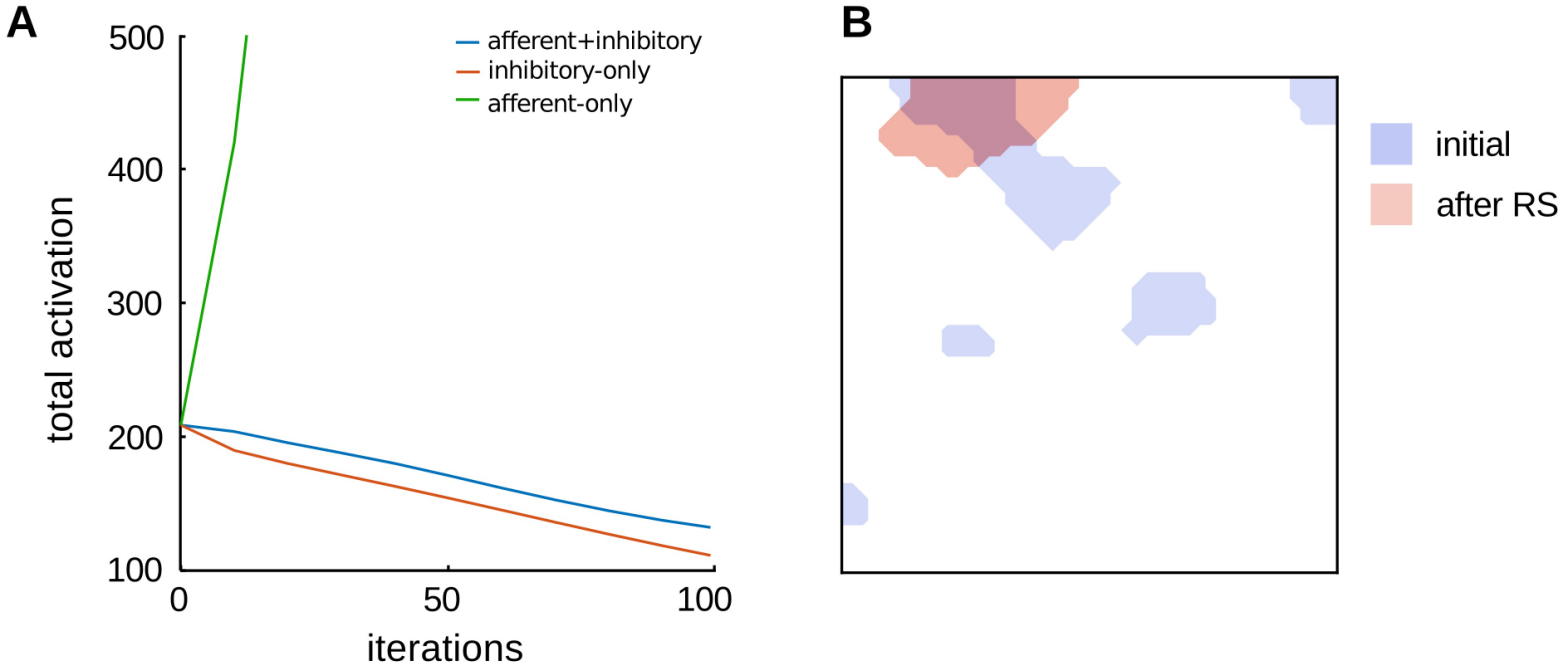
Effect of afferent and inhibitory plasticity. **A**. Comparison of the dynamics of repetition suppression in the L-model [26] in three cases: plasticity enabled in both the afferent and inhibitory connections (afferent+inhibitory, i.e., the same as in Fig 2); plasticity enabled only in the inhibitory connections (inhibitory-only); and plasticity enabled in the afferent connections, using fixed inhibitory connections (afferent-only). Plasticity in the inhibitory connections is revealed to be necessary to produce a decrease in the total activation of the network. We note that the case of afferent-only produces an increase in the total activation of the model, and that plasticity in the afferent connections alone is not guaranteed to decrease, as the activation depends on a balance between the magnitude of increase and decrease in activity of the individual units. We indeed observe both repetition suppression and enhancement of individual units, producing *sharpened* representations with repetition, as in previous work [21]. **B**. We compare the representation of the adapter stimulus in the afferent-only case before and after repetition, which shows that while the total activation in the model increases with repetition, the representation does become *sharpened* and shows a *decrease* in the number of active units.

To investigate the relative contribution of afferent versus lateral plasticity to repetition suppression, we simulated the network in three cases. In the first case plasticity was enabled in both the afferent and inhibitory connections (afferent+inhibitory, i.e., the same procedure as in Fig 2). In the second case plasticity was enabled only in the inhibitory connections, and the weights of afferent connections were kept fixed from time *t* = 0 (*inhibitory-only*). In the third case plasticity was enabled only in the afferent connections, and the weights of inhibitory connections were kept fixed from time *t* = 0 (*afferent-only*). As shown in Fig 3, the decrease in the total activation of the model cortex depends heavily on the strengthening of the inhibitory interactions between units active in the same representation, and it occurs even when afferent plasticity is disabled. However, even though the case in which inhibitory plasticity is disabled does not cause a decrease in activity, it still produces *sharpening* in the representation of the adapter stimulus (Fig 3B). Herein we use the full model with afferent and lateral plasticity enabled, while the same simulations in the other cases (inhibitory-only and afferent-only) are available as Figs C and D in S2 Text.

### Interfering representations disrupt repetition suppression

Several studies have investigated the effects of interrupting repetition suppression for an ‘adapter’ object by presenting a number of ‘intervening’ objects, and then measuring the response to the original adapter presented again. It is interesting that there exists conflicting evidence on the effect of such designs. An early study of single neurons in primate inferotemporal cortex, for example, found that repetition suppression was unaffected by the presentation of more than 150 intervening stimuli between successive presentations of the adapter pattern (Li et al., [8]). However, more recent fMRI studies with humans have reported significant differences between responses before and after interruption (Henson et al., [12, 48]). The difference between these findings might be due to a variety of factors, from differences in the measured signals (single-neuron electrophysiology versus local field potential versus functional-MRI) to differences in protocol (stimulus type, duration, task, previous exposure to the adapters etc.) and species (human versus non-human primates).

Specifically, we hypothesize here that intervening stimuli whose cortical representation overlaps significantly with that of the adapter (i.e., whose active neurons respond to both objects) may interfere with repetition suppression. Li et al., used stimuli less likely to produce overlapping cortical activations (line drawings of objects from various semantic categories), whereas the studies by Henson et al., used faces, that despite being unique and distinguishable from oneanother are processed in very localized regions of the neocortex (i.e., in the fusiform face area, FFA).

**Fig 4:**
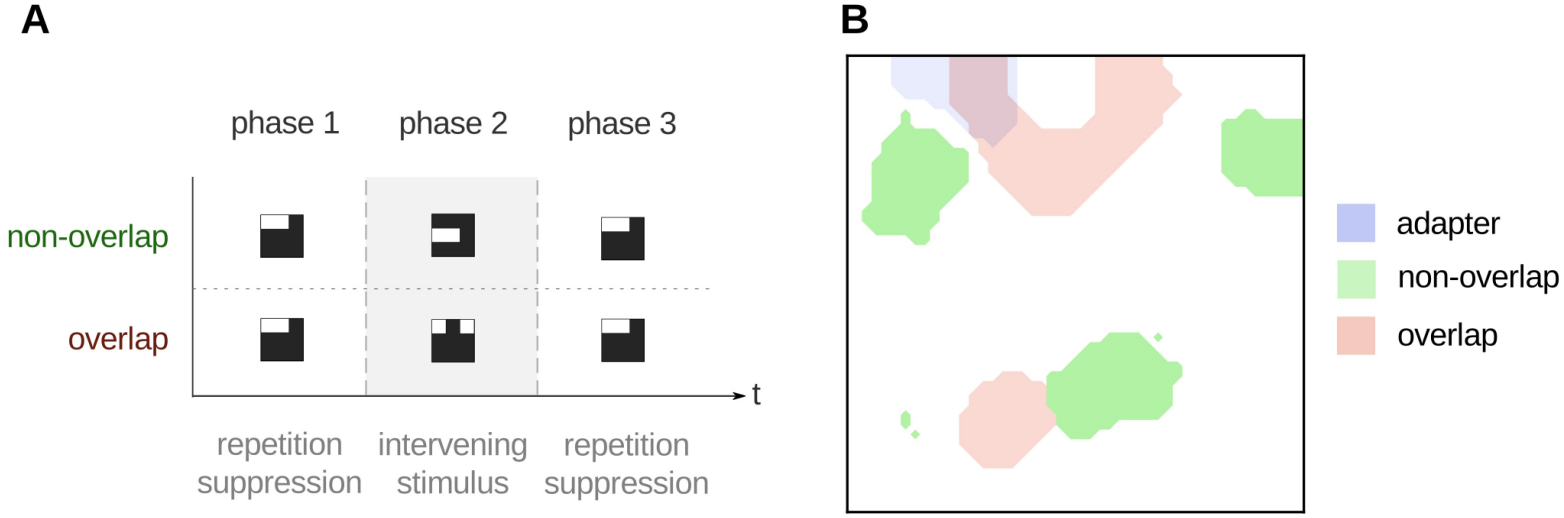
Influence of intervening stimuli on the degree of cortical overlap. **A**. The effect of intervening patterns in repetition suppression can be tested with a three phase protocol. First, an adapter object is presented to the network, in order to produce repetition suppression. In the second phase, the input is replaced with an intervening pattern (either overlap or non-overlap). Finally, the original adapter pattern is presented to the network again. Each phase consists of 100 iterations. The stimuli are nine-dimensional vectors (vizualised here as 3 by 3 grids). **B**. Comparison of the cortical representations of the phase 1 and phase 2 stimuli, computed at the end of phase 1. At this point the network has learnt an explicit representation of the adapter (blue). However, no explicit representation of the overlap or non-overlap stimuli has emerged. The intervening stimuli (phase 2) use some (‘overlap’ object; red) or none (‘non-overlap’; green) of the components of the adapter. The cortical representation to the non-overlap pattern has minimal to zero overlap with that of the adapter.

To explore these interactions further, we subjected the network to a three-phase design. In the first phase of the experiment, the adapter pattern was presented as input, thus producing repetition suppression dynamics as before. During the second phase, the network was shown a different, intervening stimulus. In the third phase, the original adapter was presented again. Each phase was run for 100 simulated steps. In what we call the ‘non-overlap’ condition, the intervening stimulus presented in phase 2 represented two ‘parts’ that did not feature in the adapter stimulus (e.g., *x*_4_ = 1 and *x*_5_ = 1). In what we call the ‘overlap’ condition, the intervening stimulus consisted of one part from the adapter stimulus and one new part (e.g., *x*_1_ = 1 and *x*_3_ = 1). The difference between these two conditions constitutes our hypothesis about the critical difference between the stimuli used by Li et al., (comparable with our ‘non-overlap’ condition) and Henson et al., (comparable with our ‘overlap’ condition); see Fig 4.

**Fig 5:**
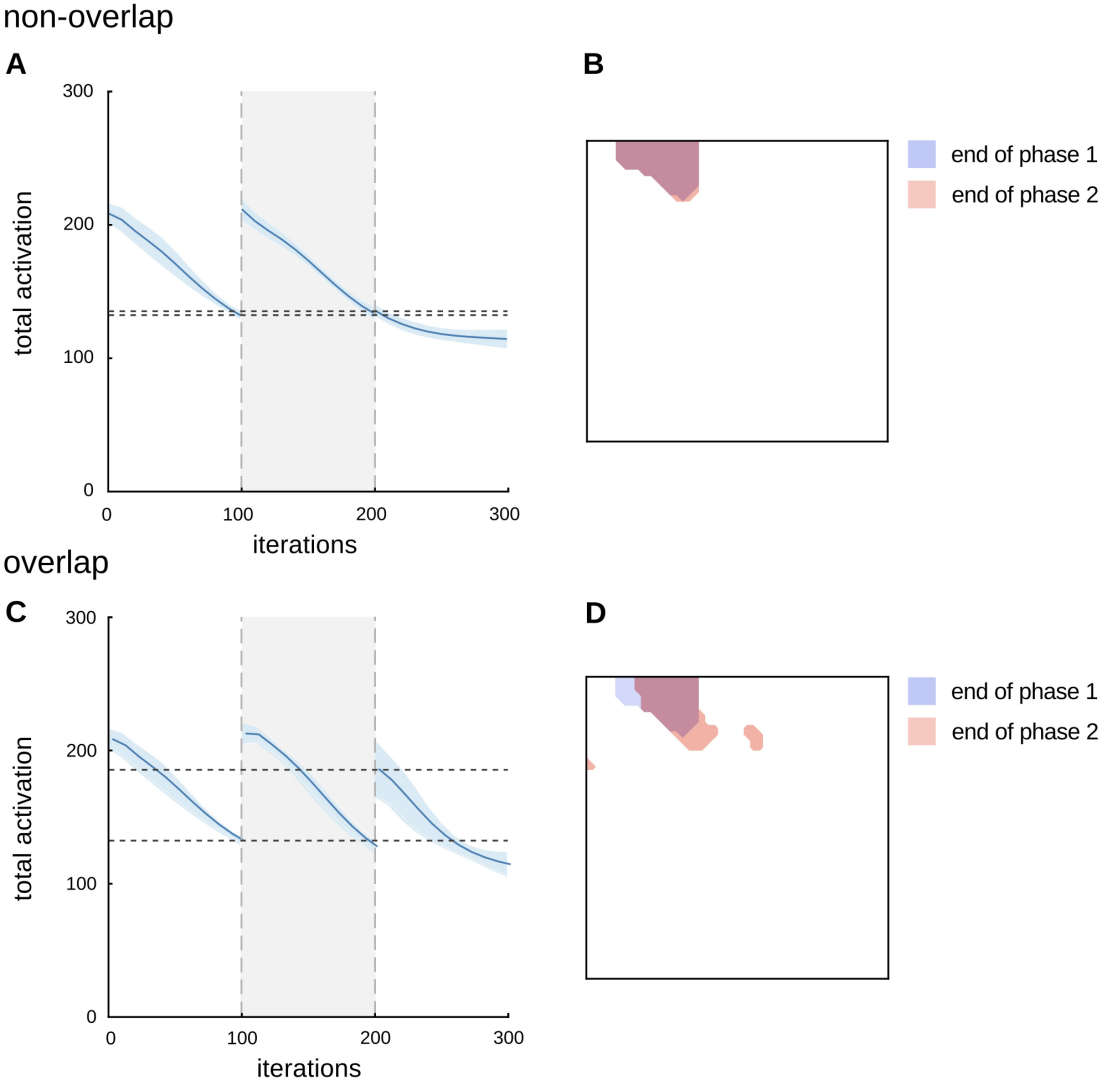
Dynamics for the non-overlap versus overlap simulations. **A**. The level of activity in the network is not affected by an intermediate phase in which a different (non-overlap) stimulus is presented. The plot shows the model activity averaged over 10 simulations pre-trained with different random initial conditions. The shaded area is the standard deviation. **B**. The cortical representation of the adapter does not change during the intermediate phase (iterations 100 to 200) when presented with the non-overlap stimulus. Indeed, there is no interaction between the representation of the adapter and intervening stimuli. **C**. The activity generated by the model is different when the overlap stimulus is used instead of the non-overlap stimulus. After the intermediate phase, the activity increases rather than remaining constant (as it does in panel A). **D**. The representation of the adapter stimulus changes during the intermediate phase when the overlap stimulus is used. Note that the total activation computed in panels A and C is the sum of the activity of all units (not the number of active units shown in the cortical representation; B,D).

**Fig 6:**
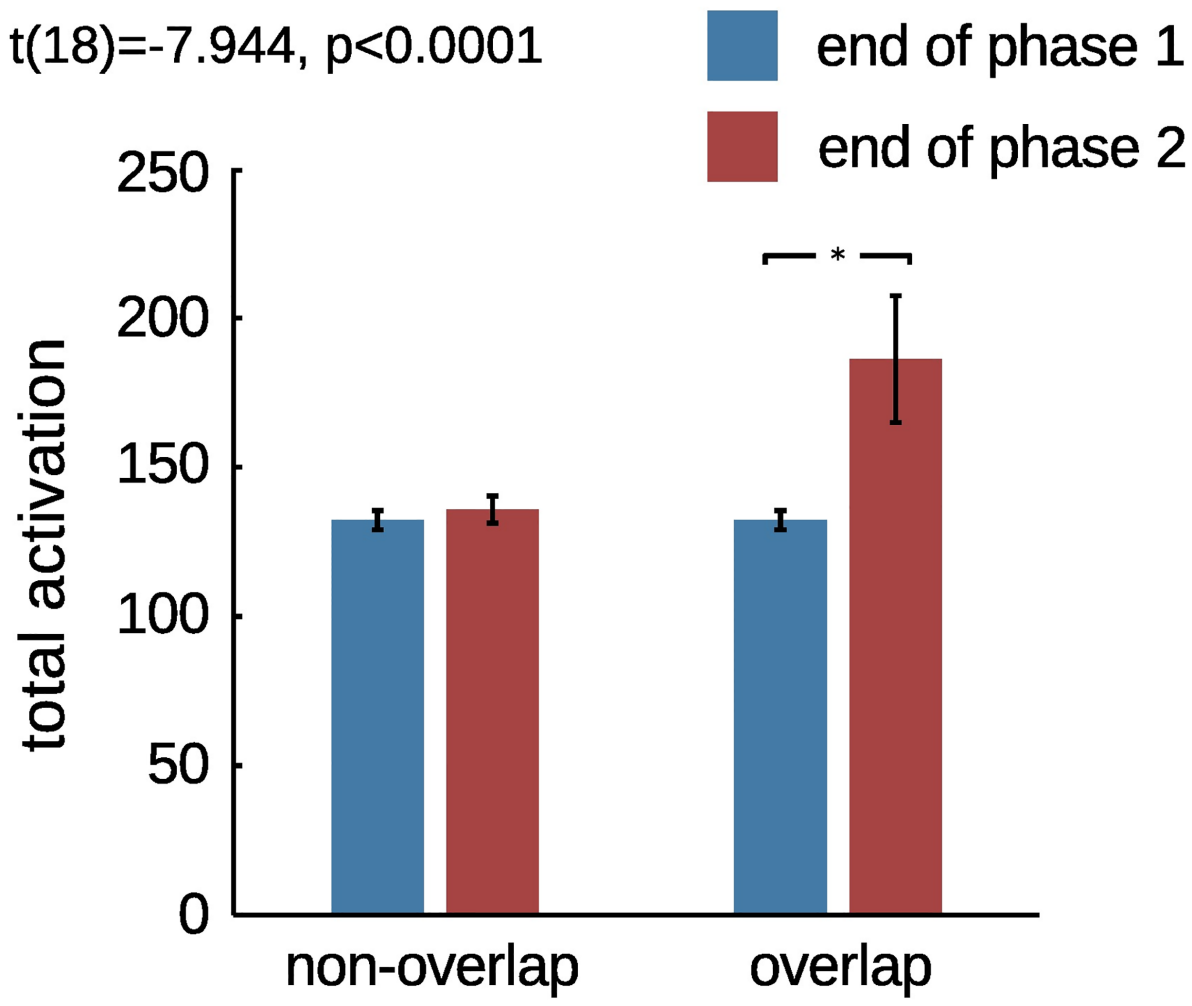
Non-overlap versus overlap. Comparison of the difference in activity produced by the adapter stimulus before and after the intermediate phase (iteration 100 versus 200), in the non-overlap and overlap simulations. The difference due to the overlap stimulus was statistically significant (paired t-test, *t*(18) = −7.944, *p* < 0.0001). Any difference due to the non-overlap stimulus was not significant (paired t-test, *t*(18) = −2.0161, *p* > 0.05). The tests were performed on data from 10 different simulations, using models pre-trained and ran with different random seeds.

In the non-overlap condition, repetition suppression was not affected by presentation of the phase 2 stimulus (Fig 5A), and the cortical response to the adapter did not change between the final trial of phase 1 and the first trial of phase 3 (Fig 5B). The network response in the non-overlap condition is therefore consistent with the findings of Li et al. [8]. In the overlap condition, however, repetition suppression was strongly affected by the presentation of the intervening stimulus (Fig 5C), which caused an increase in the response to the adapter at the beginning of phase 3, reflecting a re-organization of the representation of the adapter (Fig 5D). A comparison presented in Fig 6 reveals no significant difference in the total cortical response to the adapter before versus after phase 2 in the non-overlap condition (paired t-test, *t*(18) = −2.0161, *p* > 0.05), but a significant difference between the two responses in the overlap condition (paired t-test, *t*(18) = −7.944, *p* < 0.0001). Statistical tests were performed on data from 10 independent simulations, pre-trained and run with different random seeds and random initial conditions.

## Discussion

The self-organising models of Stevens and colleagues [26] constitutes our current best theory of the emergence of topological maps in primate neocortex. The distinguishing feature of this theory is that both afferent and lateral connectivity is updated using mechanisms of Hebbian plasticity. As a consequence, intra-cortical interactions strengthen between units that are co-active. In particular, repeated presentations of the same stimulus produce a strengthening of the inhibitory interactions between the units that are recruited into its cortical representation (i.e., the units activated by the presentation of the stimulus), and thus a lowering of the overall level of activity. We suggest that such factors might underlie the phenomenon of repetition suppression.

A previous model has shown that sharpening can arise from plasticity in afferent projections alone, if strong competition between the model units is present [21]. However, repetition suppression was only measured in individual units, and the authors explain that the overall activation in the model is not guaranteed to decrease with repetition, as it depends on a balance between the magnitudes of suppression and enhancement of individual units. Further, this model approximated the net effects of recurrent inhibitory interactions in the neocortex using a simple winner-take-all operation, which may only account for few of the complex interactions that emerge from plasticity in real biological networks. In this study we explicitly simulated the recurrent cortical interactions that mediate local competition, and we showed that plasticity in the lateral inhibition between cortical units is sufficient to account for repetition suppression, even without afferent plasticity (Fig 3). Our main simulations include both lateral and afferent plasticity, hence the present results do not challenge the idea that afferent plasticity contributes substantially to repetition suppression. Instead, we claim that repetition suppression reflects a combination of both afferent and lateral plasticity.

This account is broadly consistent with sharpening theory, according to which a reduction in the cortical response reflects a narrowing of tuning curves and therefore an increase in the selectivity of neuronal activity. The current model extends sharpening theory by emphasising also the role of intra-cortical plasticity. According to this extended ‘inhibitory sharpening’ model, tuning curves narrow due to the effects of both afferent and lateral plasticity. Essentially, the co-activation of units recruited in the representation of the adapter stimulus causes a strengthening of mutual inhibition between them via Hebbian plasticity, and as this mutual inhibition builds over time the responses of individual units become more selective, the overall cortical response decreases, and the least selective neurons are silenced.

Alternatives to the sharpening theory are theories based on neural fatigue, according to which repetition suppression reflects a depletion in the resources required by neurons in order to spike [8, 20]. Neural fatigue theories seem to be supported by single-unit studies showing that the greatest reduction in cortical activity is attributable to the neurons that respond most strongly to the first presentation of an adapter stimulus. However, the inhibitory sharpening account provides an alternative explanation. By Hebbian association, the units that happen to be most active upon first presentation of the adapter stimulus subsequently develop the strongest mutual inhibition.

We further note that the dependency of the inhibitory sharpening theory on plastic lateral connectivity makes its dynamics consistent with the predictive coding framework, which also offers an alternative interpretation of repetition suppression compared with theories based on neural fatigue and sharpening [49–51]. According to predictive coding, each cortical area predicts the incoming sensory signal, and makes the unpredicted portion of the signal (the prediction error) available to subsequent processing areas. Repeated presentation of a stimulus leads to synaptic changes that improve the ability to predict future stimuli, reducing the prediction error and thus reducing levels of cortical activation [52, 53].

Interestingly, when a neural mass model of cortical dynamics was inverted to fit empirical data, the assumption of an intrinsic (intra-area) and extrinsic (inter-area) cortical connectivity which reduced exponentially with stimulus presentations could explain most of the suppression (though an additional phasic term helped increase the fit) [54]. The authors interpreted the changes in intrinsic and extrinsic cortical circuitry in terms of the perceptual and plastic components of the computations required for predictive coding. Specifically, they reported a consistent decrease in coupling in the intrinsic connectivity following the first stimulus presentation, which is broadly consistent with the Hebbian buildup of recurrent lateral inhibition predicted by the present model. An extension of the present model to include extrinsic interactions between cortical areas, guided by the predictive coding framework, may allow for a mechanistic account of the contribution of plastic recurrent cortical interactions to hierarchical cortical computation. Moreover, as the cortical interactions simulated in the present model are known to subserve topological map formation, this approach could provide a theoretical bridge between predictive coding (acting on psychophysical timescales) and map development (acting on developmental timescales).

Other avenues for future research include establishing the relationship between inhibitory sharpening and accounts of repetition suppression in terms of increasing speed of processing [55] and enhanced neural synchronization [14].

The critical test of our model, and hence of the extended ‘inhibitory sharpening’ theory that it represents, is demonstrated in Figs 5 and 6. Experimental confirmation of the prediction that repetition suppression may be modulated and disrupted by stimuli with a cortical representation that overlaps that of the adapter (e.g., comprising a subset of the features of the adapter stimulus), would constitute support for the inhibitory sharpening theory. In contrast, in the same protocol neural fatigue would likely predict a further decrease in cortical activity, as the shared units would undergo further repetition suppression independently in each of the three phases. Sharpening would also predict a further suppression of the activity due to the overlap stimulus, but the decrease could be minimal or absent depending on the narrowing of the tuning of the neurons selective to the adapter stimulus after its first repetition. Predictive coding, on the other hand, might exhibit more complex dynamics. Indeed, the presentation of the overlap stimulus could introduce a new statistical co-occurrence between the parts/features shared by the adapter and the overlap stimuli, and the novel parts of the overlap stimulus. Such co-occurrence would not be observed on the second repetition of the adapter, in the third phase of the protocol, which would result in an increased error due to the un-predicted mismatch and thus an increase in activity similar to that from inhibitory sharpening. This is however not too surprising, as plastic recurrent lateral connections can learn the statistical co-occurrence of features [30, 46, 56].

**Fig 7:**
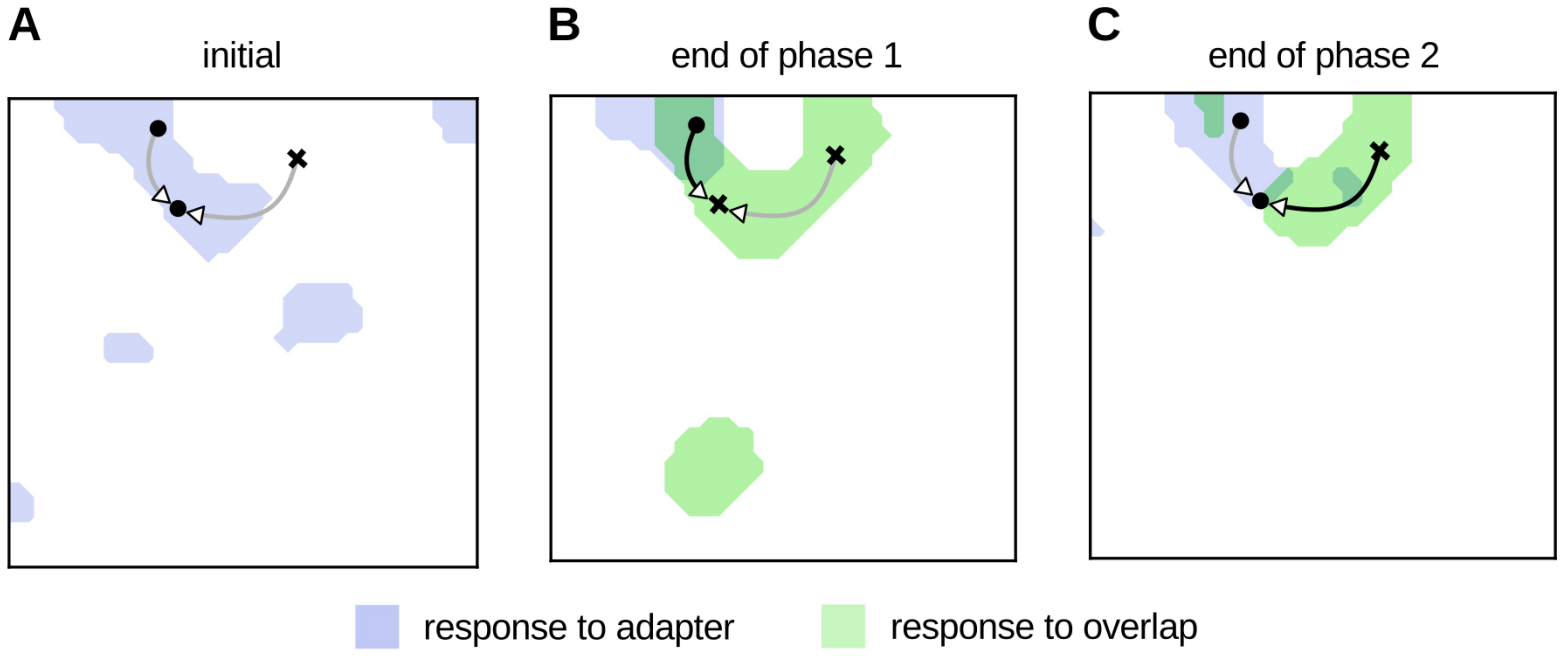
Changes in lateral connectivity underlying repetition suppression. This figure exemplifies the changes in the effective contribution of four different inhibitory connections from the same pre-synaptic neuron during each experimental phase. The width and color of the lines indicates the strength of inhibition received by the post-synaptic units; the product of the pre-synaptic activity and the weight of the inhibition. Each panel shows the cortical representation of the adapter and overlap stimuli, on the first iteration of each phase. **A**; iteration 0, **B**; iteration 100, and **C**; iteration 200. During the first phase the strength of the inhibition between the co-active units (i.e., those belonging to the same representation) increases, leading to a subset of them being suppressed. In the intermediate phase, however, the inhibition between the shared units (overlap/adapt) and the units selective only to the overlap pattern increased, and further led to some of the shared units to be removed from the representation of the adapter in favor of the overlap stimulus. Thus, some of the units in the representation of the adapter became suppressed, while others shifted the distribution of their inhibitory inputs to units in the representation of the overlap stimulus. When the adapter was presented again, the total amount of inhibition that the units that had been suppressed during the first phase received was reduced, as either the inhibitory weights between units in that representation had decreased or the units that inhibited them were not active anymore. Another example is shown in Fig F in S2 Text.

To understand why the L-model predicts an increase in activation after the intermediate phase, it is useful to look at the changes in the representations of the stimuli before and after each of the three phases (Fig 7). During the first phase of repetition suppression, the co-activation of units recruited in the representation of the adapter stimulus led to a strengthening of their mutual inhibition by Hebbian plasticity, and thus to a suppression of responses in a subset of units (Fig 2B). However, presentation of a second stimulus sharing features of the adapter increased the inhibition between units selective only to the second stimulus and units responding to both, and further led to some of the units responsive to both to drop out of the representation of the adapter stimulus and into the representation of the second stimulus. Thus, some of the units in the representation of the adapter were suppressed, while others had the distribution of their inhibitory inputs shifted towards units selective for the second stimulus (see Figs E and F in S2 Text), due in part to divisive normalization of the synaptic weights (Eq 2). When the adapter pattern was presented again in a third phase, the total inhibition received by the units suppressed during the first phase was reduced. This is because either the inhibitory weights between units representing the adapter had decreased or the inhibiting units were no longer active. The variety of inhibitory interactions is illustrated in Fig 7, which shows the change in the influence of one pre-synaptic unit over four post-synaptic units. Another example is shown in Fig F in S2 Text.

The model can account for why some studies have found that repetition suppression is affected by intervening patterns whereas others have not, in terms of differences in the choice of the stimuli. In particular, Li and colleagues [8] used stimuli that were sufficiently different from oneanother, which could therefore have produced cortical responses with little overlap and hence little interference. Henson et al. [12, 48], on the other hand, used pictures of faces (famous versus unfamiliar), that despite their individual differences could have elicited overlapping representations whose effect would have been further amplified by the large number of intervening stimuli (around 100). In support of our account of these effects, we note that Sawamura and colleagues [57] found that the firing rates of neurons in monkey infero-temporal cortex depend on whether a preceding stimulus was the same (causing repetition suppression), different but capable of making the same neuron fire (causing a response similar to the prediction in our overlap condition), or different and not capable of making the neuron fire (causing a response similar to the prediction in our non-overlap condition).

**Fig 8:**
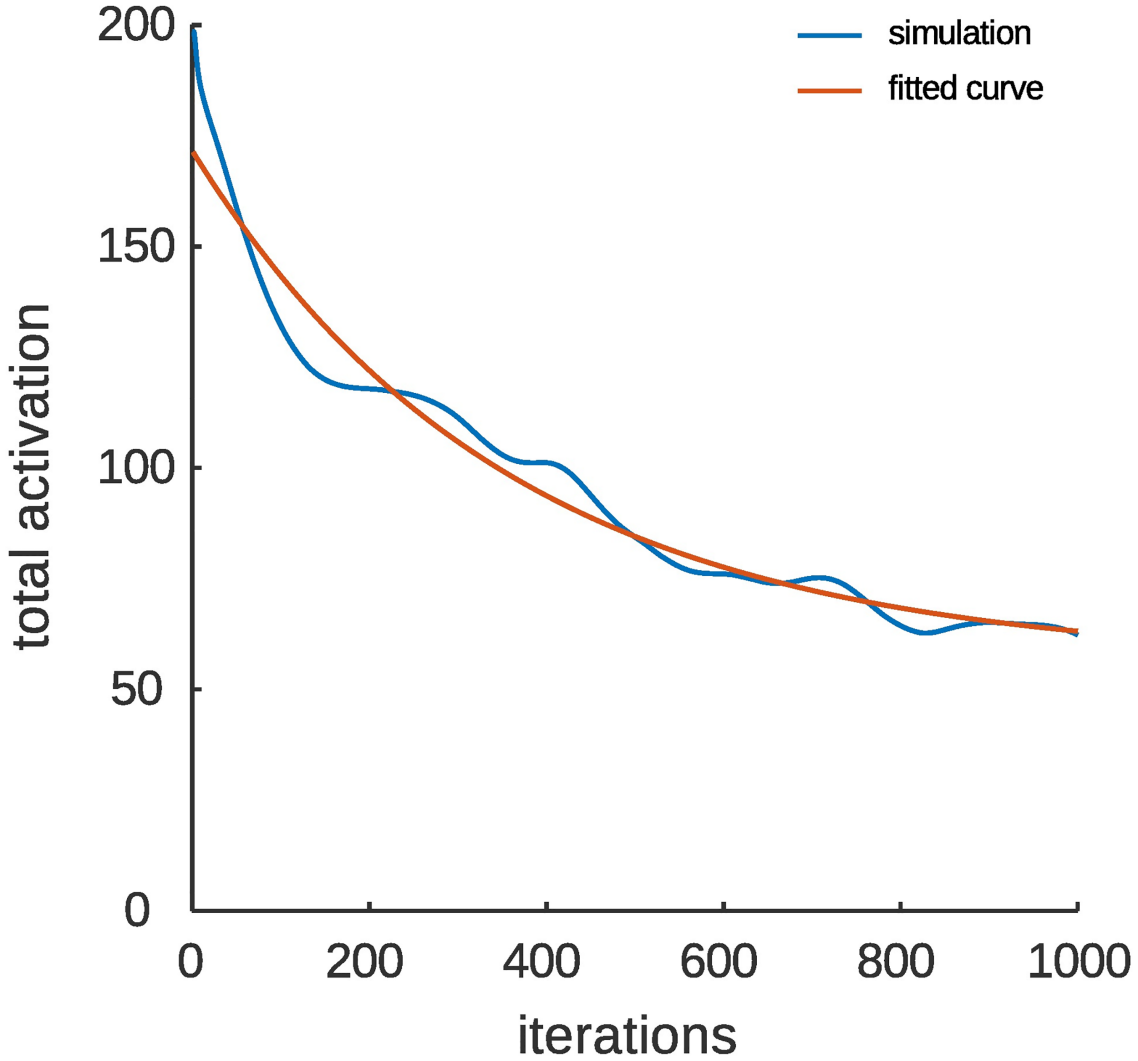
Longer term dynamics of repetition suppression. Repetition suppression in the model was simulated for an extended period of 1000 iterations. The longer-term dynamics conform to an exponential decay fit to the total activation (*y*(*t*) = *ae*^−*bt*^ + *c*), approximating the general form of the dynamics of repetition suppression measured by e.g., [8, 58].

A limitation of the current modelling framework is that due to the discrete timescales of the settling steps of the recurrent dynamics, and of the onset of new iterations, neurophysiological timescales in the model are difficult to reconcile precisely with psychological timescales. A single presentation of a stimulus on a psychologically relevant timescale corresponds to multiple simulated ‘iterations’ of the model. To reconcile stimulus presentations and model iterations approximately, we ran the model for an extended period of 1000 iterations. The longer-term dynamics conformed to an exponential decay fit to the total activation (*y*(*t*) = *ae*^−*bt*^ + *c*), and thus match the empirically observed dynamics of repetition suppression [8, 58]. Although there is significant variability between studies of repetition suppression regarding the number of repetitions after which activation plateaus, which may depend on differences in protocol, species and recording techniques, the various estimates in the literature agree broadly that a plateau is reached within 5–10 repetitions. Thus, drawing a parallel to the fitted exponential curve in Fig 8, which reaches a plateau within the 1000 iterations displayed, it may be possible to consider the 100 iterations used throughout this manuscript as roughly corresponding to a single repetition of the adapter stimulus.

The mechanistic account of repetition suppression (and facilitation) offered by the inhibitory sharpening theory may be challenged further by investigating the effects of interference in perceptual discrimination tasks, using the degree of similarity and overlap in cortical representations to quantify the ‘confusion’ between similar stimuli. For example, multi-voxel fMRI analysis such as representational similarity analysis [59, 60], could be used to measure the similarity and overlap between representations, and multi-voxel pattern analysis [61] could be used to correlate the performance of a classifier built on the representations of the stimuli (measured by fMRI imaging) with the behavioral discrimination accuracy.

Experimental confirmation of the predictions of the model would provide evidence that repetition suppression, as an emergent property of plastic lateral interations, reflects a transition in the cortical representation of stimuli from a distributed to a localist encoding.

## Supporting information

S1 Text Supplementary materials and methods. Table of parameters and description of the homeostatic mechanisms used in pre-training.
(PDF)

S2 Text Supplementary results. Exploration of the limit cases in which plasticity is restricted to either the afferent or inhibitory interactions in the model.
(PDF)
