# Supplementary material for: Familiarization: A theory of repetition suppression predicts interference between overlapping cortical representations
Source: PLoS One. 2017 Jun 12;12(6):e0179306. doi: 10.1371/journal.pone.0179306 (PMC5467900; doi:10.1371/journal.pone.0179306)
Supplement: S1 Text — Table of parameters and description of the homeostatic mechanisms used in pre-training. (PDF) [file pone.0179306.s001.pdf]

Familiarization: a theory of repetition suppression predicts interference between overlapping cortical representations

Giacomo Spigler, Stuart P. Wilson

S1. Supplementary Materials and Methods

Pre-training of the network was performed with the addition of homeostatic plasticity. This mechanism is used to compute the dynamic threshold (θ) of the piecewise-linear output function σ, which distributes activity evenly across the network and thus leads to stable map development [Stevens et al., 2013].

To update the threshold, a smoothed exponential average of the settled activity of each model unit is recorded as

$$\overline{\eta_j}(t) = (1-\beta)\eta_j(t) + \beta\overline{\eta_j}(t-1)$$

where β is referred to as a smoothing parameter.

At the end of each iteration, the dynamic threshold is updated as

$$\theta(t) = \theta(t-1) + \lambda(\overline{\eta_j}(t) - \mu)$$

where λ is the homeostatic learning rate, and μ is the target average activity.

The table below shows the parameters used for the simulations. The learning rates represent the total amount of change across all the model units. The learning rate of each unit is computed as $\epsilon = \frac{\epsilon_p}{N_{units}}$, where N_{units} is the number of units in the network ($N_{units} = 48 \cdot 48$).

Parameter	Value
# Units	$48 \cdot 48$
Afferent Strength (α_A)	2.2
Excitatory Strength (α_E)	1.2
Inhibitory Strength (α_I)	2.3
Afferent Learning Rate (ϵ_A)	0.1
Excitatory Learning Rate (ϵ_E)	0.0
Inhibitory Learning Rate (ϵ_I)	0.3

Parameters used to pre-train the models.

Parameter	Value
Afferent Strength (α_A)	1.5
Excitatory Strength (α_E)	1.2
Inhibitory Strength (α_I)	2.3
Homeostatic learning rate (λ)	0.01
Homeostatic smoothing (β)	0.991
Homeostatic initial average ($\overline{\eta_j}(0)$)	0.15
Homeostatic target activity (μ)	0.024
