# Supplementary material for: Familiarization: A theory of repetition suppression predicts interference between overlapping cortical representations
Source: PLoS One. 2017 Jun 12;12(6):e0179306. doi: 10.1371/journal.pone.0179306 (PMC5467900; doi:10.1371/journal.pone.0179306)
Supplement: S2 Text — Exploration of the limit cases in which plasticity is restricted to either the afferent or inhibitory interactions in the model. (PDF) [file pone.0179306.s002.pdf]

Familiarization: a theory of repetition suppression predicts interference between overlapping cortical representations

Giacomo Spigler, Stuart P. Wilson

S2. Supplementary Results

Role of plasticity in the afferent and inhibitory interactions

Here we compare the effects of plasticity in the afferent and inhibitory interactions in the model by setting the learning rate of either one of the connection types to zero, and then replicating the three-phase protocol discussed in the main text. The comparison is reported in Fig. C (inhibitory-only) and Fig. D (afferent-only). We observe that the two models exhibit opposite dynamics after the intermediate phase, with a predicted *increase* in activity in the inhibitory-only case, similarly to the results from the combined model, and a further *decrease* in the afferent-only simulations. However, both models show a spreading of the representation of the adapter stimulus after the intermediate phase, as an un-sharpening of its representation. This is interesting as the afferent-only model shows a similar behavior in the first phase, producing sharpening (as seen from the changes in the model representation, see Fig. 4B in the main text), that is not accompanied by a decrease in the total activity. Indeed, while afferent plasticity continually improves the tuning of the model units to the adapter, thus increasing their activation, the lack of lateral inhibitory plasticity means that units that are active when the adapter is presented, but whose preferred features were not co-occurring before its presentation, have no means to inhibit each other and thus to compensate the increase in activity. This limitation, however, might not apply to other models based on fixed inhibition between the units, for example in [Norman et al., 2003], where inhibition is modelled with a k-WTA operation uniform across all the units.

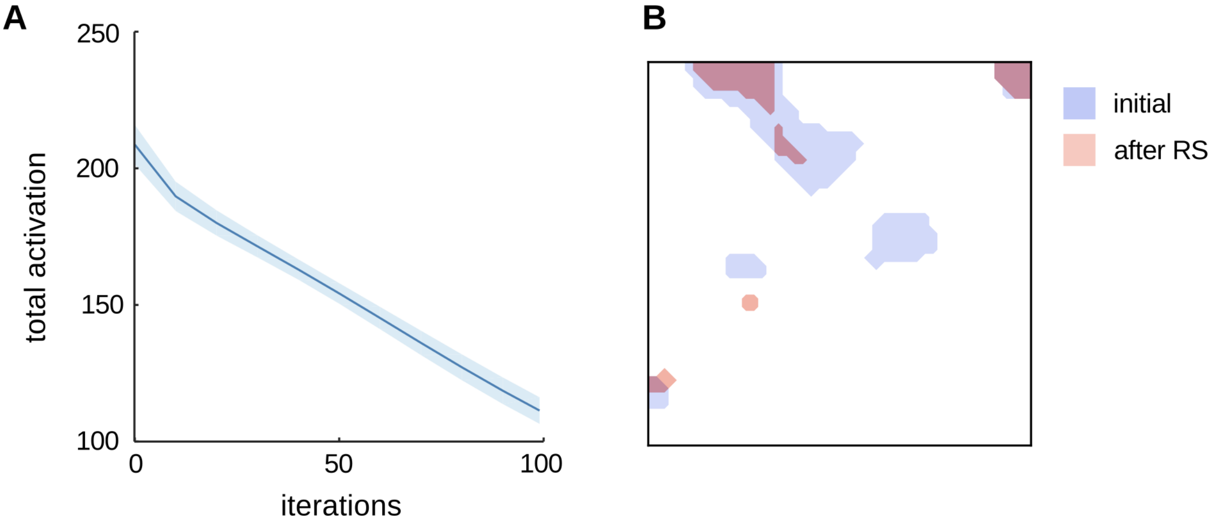

Figure A. Simulations showing repetition suppression where the lateral inhibitory connections are the only plastic connections in the simulation (i.e., plasticity in the afferent connections is set to zero). This Figure is analogous to Fig. 2 in the main text. **A**. Repetition suppression dynamics when repeatedly presented with the same input stimulus. The total activation is computed at each simulated timestep as the sum of the activity of all units in the network. The plot is an average of 10 simulations ran with different random initial conditions, with the shaded area representing standard deviation. **B**. The cortical representation of the repeated stimulus is visualized by thresholding the activity of the network before (blue) and after (red) repetition suppression. Representations produced by the model are distributed across stable blobs of highly active units. After repetition suppression, the response is “sharpened”, i.e., the sizes of blobs of super-threshold activity shrink.

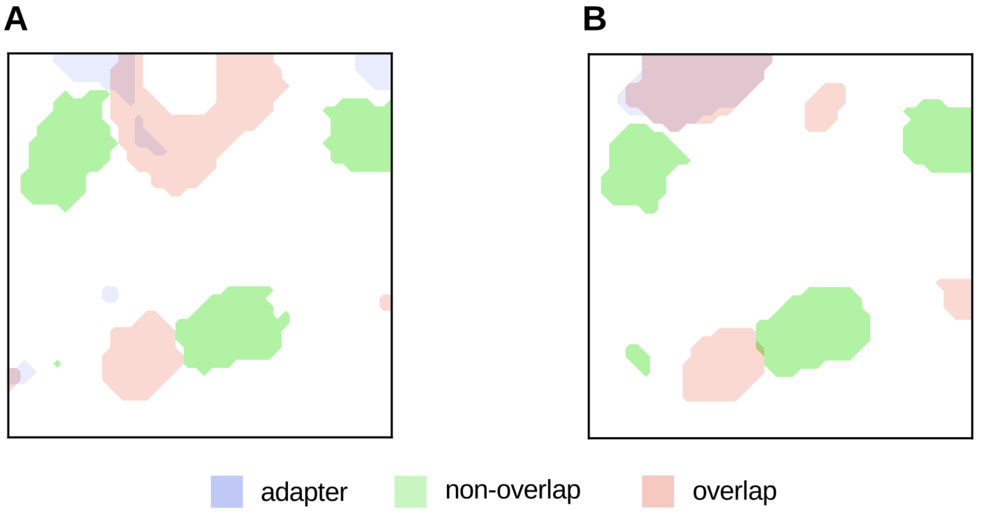

Figure B. Comparable with Fig. 5 **B** in the main text. The cortical representations of the Adapt and Overlap patterns overlap, while those of Adapt and Non-Overlap do not. **A**. Representations computed in the inhibitory-only model. **B**. Representations computed in the afferent-only model.

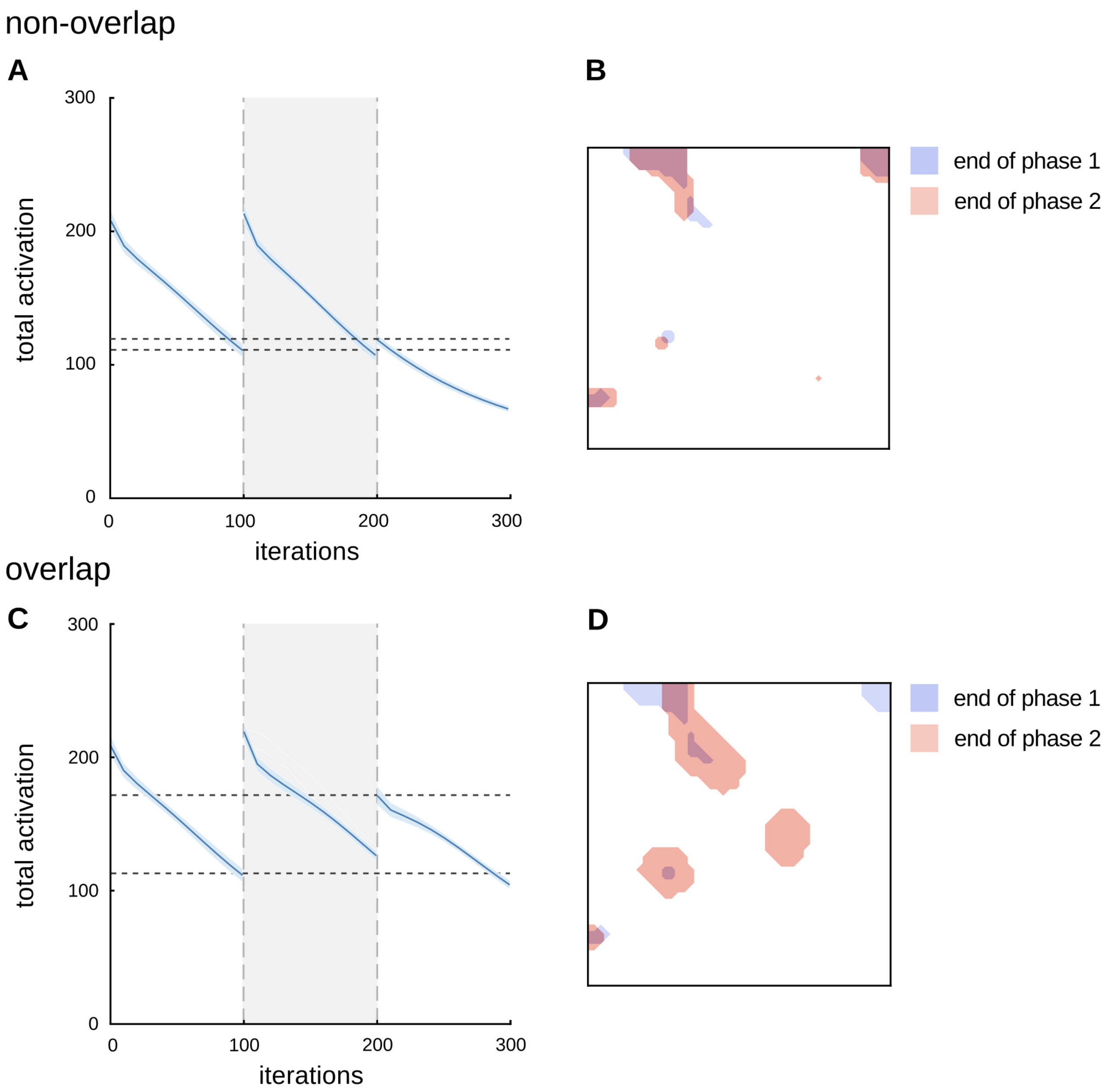

Figure C. **A-B**. Comparable with Fig. 6 (**A-B**), in the case where only the lateral inhibitory connections are plastic. Intervening stimuli whose cortical representations do not overlap with that of the adapter do not affect its repetition suppression dynamics. **B-C**. As in Fig. 6 (**C-D**). Intervening stimuli whose cortical representations overlap with that of the adapter producee interference.

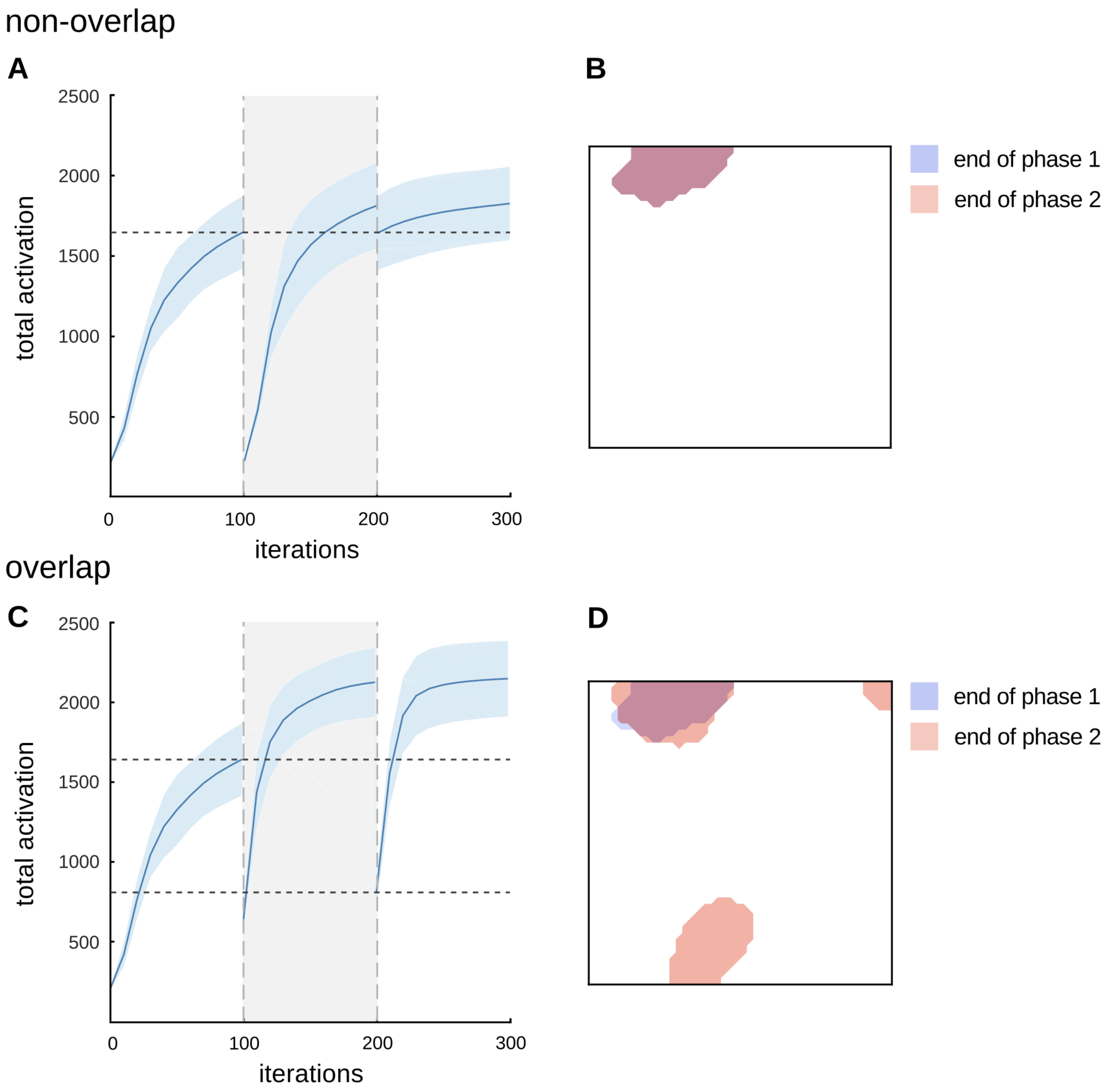

Figure D. **A-B**. Comparable with Fig. 6 **(A-B)**, in the case where only the afferent connections are plastic. Intervening stimuli whose cortical representations do not overlap with that of the adapter do not affect its repetition suppression dynamics. **B-C**. As in Fig. 6 **(C-D)**. Intervening stimuli whose cortical representations overlap with that of the adapter producee interference. It is interesting to observe that while the average activity after the intermediate phase is lower than before, the number of active units increases, as in the case in which plasticity is enabled in the lateral inhibitory connections.

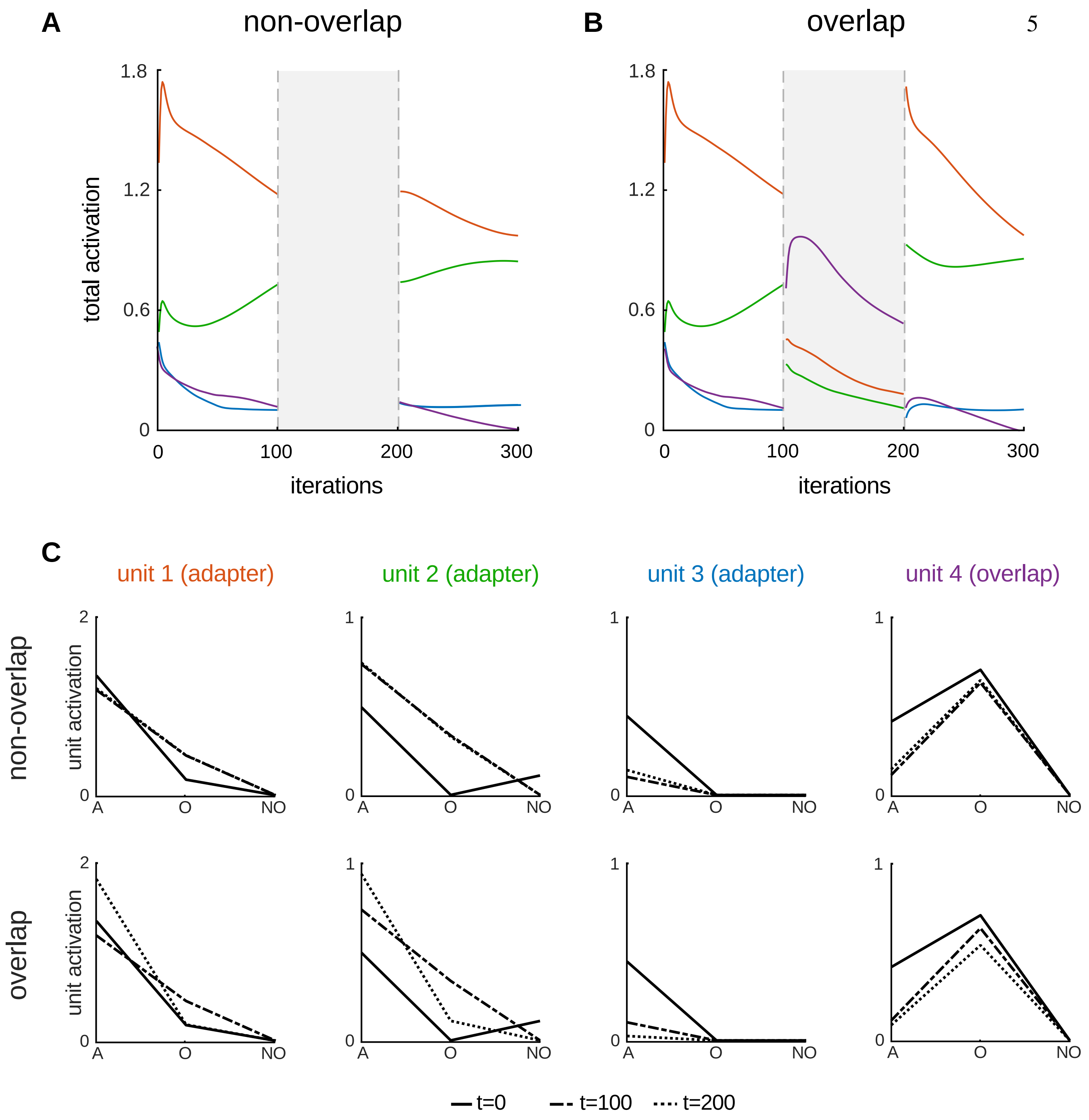

Figure E. **A-B**. Activation of three units in the model in the two conditions (**A** *non-overlap*, and **B** *overlap*) in the combined model (afferent+inhibitory). The red and blue units show repetition suppression, with the blue unit reaching a minimum value in the first phase of the protocol. The green unit exhibits repetition enhancement dynamics. The red and green units show selectivity for the overlap stimulus, and they are both suppressed during the intermediate phase. The purple unit is more selective to the intervening/overlap stimulus. **C**. Tuning curves of the three units with a fourth that is selective to the overlap stimulus, in both the non-overlap and overlap conditions, at the beginning of the three phases ($t = 0, t = 100, t = 200$). The tuning curves were computed by presenting the three stimuli **A**-adapt, **O**-overlap and **NO**-non-overlap and allowing the dynamics of the network network to settle. The units 1, 3 and 4 exhibit repetition suppression, while unit 2 shows enhancement. It is interesting to observe that, in contrast to the sharpening theory, units 1 and 2 actually broaden their tuning during the first phase. This is due to a dis-inhibition of the units shared in the representations of the stimuli. Indeed, the presentation of the adapter leads to a strengthening of the inhibitory interactions from the units that are active in its representation, which in turn produces a decrease in the strength of those originating from units active only in the representation of the overlap stimulus, by means of weight normalization. The effect of dis-inhibition is further supported by the decrease in activity with repetition by units that responded strongly to the adapter. It is interesting to note that this broadening of the tuning curves has been found experimentally (e.g., macaque area MT [Kar and Krekelberg, (2016)]) and has been investigated in computer models of the primary visual cortex based on recurrent inhibition [Teich and Qian, 2003]. In any case, we observe sharpening in the intermediate phase for units 1, 2 and 4, with a stronger decrease in response for the less preferred stimuli.

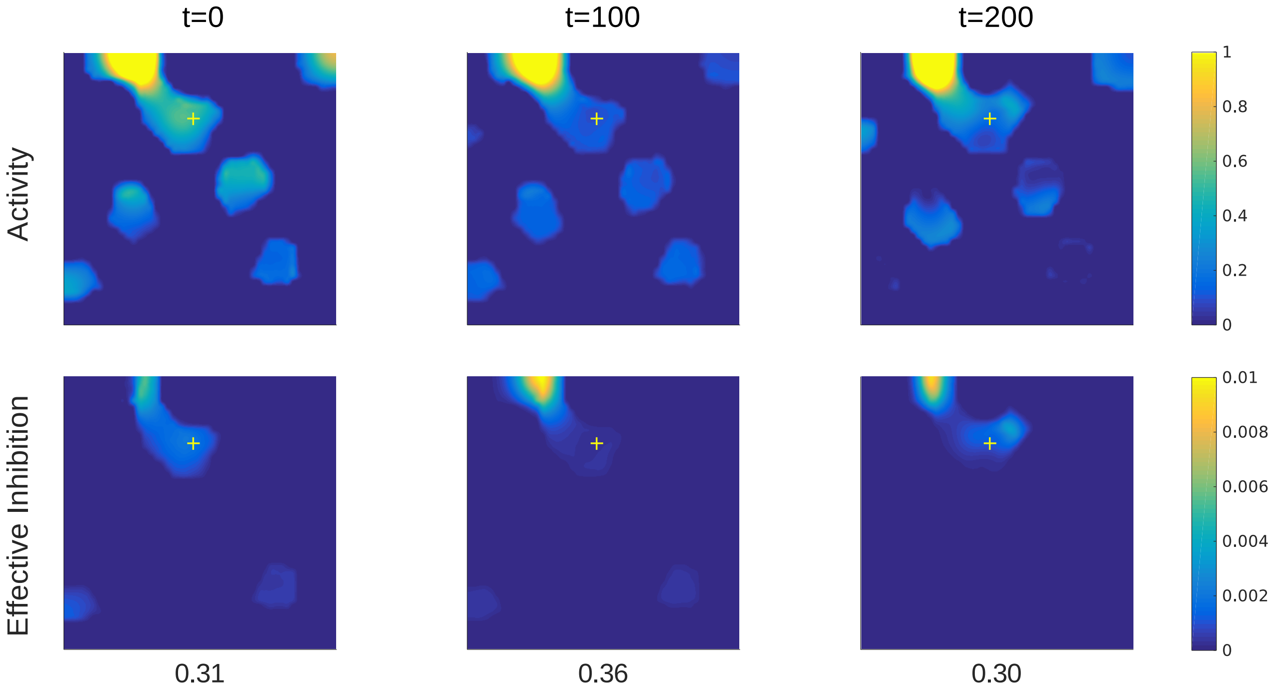

Figure F. A snapshot of the activation produced by the combined model (afferent+inhibitory) at the beginning of the three phases in the overlap condition (top row), together with the effective inhibition received by the unit marked with a cross (bottom row). Effective inhibition is computed as the product of the strength of the inhibitory connection between the unit marked with a cross and each other unit, and the activation of the second unit. Below each plot is the value of the total effective inhibition received by the marked unit (i.e., 0.31 at the beginning of the simulations, 0.36 after the first phase of repetition suppression, and 0.30 after the intermediate/overlap phase). As discussed in the main text, the L-model produces an increase in inhibition between the units active in the representation of the adapter (i.e., here from 0.31 to 0.36). The presentation of the overlap stimulus, however, shifts the inhibitory weights of the units active in both the representation of the adapter and overlap stimuli to the units active in the representation of the overlap stimulus only, thus reducing the amount of inhibition that the adapter-selective units receive when the adapter is presented again (phase 3) and leading to an increase in their activity.
